# Tobacco Smoke Exposure during Childhood: Effect on Cochlear Physiology

**DOI:** 10.3390/ijerph10115257

**Published:** 2013-10-24

**Authors:** Alessandra S. Durante, Beatriz Pucci, Nicolly Gudayol, Beatriz Massa, Marcella Gameiro, Cristiane Lopes

**Affiliations:** 1 Faculdade de Ciências Médicas da Santa Casa de São Paulo (FCMSCSP, School of Medical Sciences of Santa Casa of São Paulo), Arnaldo Vieira de Carvalho Foundation, Street Dr. Cesário Mota Júnior, 61 São Paulo 01221-020, Brazil; E-Mails: beatrizpmassa@hotmail.com (B.M.); marcella_113@hotmail.com (M.G.); cristiane.lopes@fcmsantacasasp.edu.br (C.L.); 2 Irmandade da Santa Casa de Misericórdia de São Paulo (ISCMSP, Santa Casa Sisters of Mercy Hospital of São Paulo), Street Dr. Cesário Mota Júnior, 112, São Paulo 01221-020, Brazil; E-Mails: beatrizpcpucci@gmail.com (B.P.); nihgudayol@yahoo.com.br (N.G.)

**Keywords:** outer hair cells, spontaneous otoacoustic emissions, cochlea, growth and development, smoking, child, tobacco

## Abstract

The rate of smoking in Brazil is about 18.8%. Exposure to environmental tobacco smoke is one of the major factors predisposing children to several hazardous health problems. The objective of the present research was to analyze the effect of tobacco smoke exposure during childhood on cochlear physiology by measuring the transient evoked otoacoustic emissions (TEOAE) response levels. Cotinine, the main metabolite of nicotine, was measured in 145 students’ (8–10 years old) urine. Sixty students indicated tobacco smoke exposure (TSE) (cotinine urine levels ≥ 5.0 ng/mL) and 85 did not. The evaluation of TEOAE of TSE students showed lower response levels, mainly on frequencies of 2.8 kHz on the right and left ears and 2.0 kHz on left ear and lower signal noise response levels, mainly on the 1.0 kHz and 1.4 kHz frequencies, when compared to controls that were not exposed to tobacco. The mean hearing loss in tobacco smoke exposure children was 2.1 dB SPL. These results have important implications on the damage to the cochlear structures and indicate a possible loss in hearing and hearing ability development.

## 1. Introduction

During the last 20 years, Brazil has introduced tobacco control policies, such as cigarette-specific taxes, warnings on cigarette packages, smoke-free air laws, bans on tobacco marketing practices, *etc*. In the same period, the smoking rates have fallen by 50% [1]. Recent available data shows that the smoking rate in Brazil is about 18.8%, being 22.7% among men and 16% among women [2].

Despite the improvements on public health due to tobacco control policies in Brazil, the bad impact of tobacco smoke on people that smoke still remains. Furthermore these policies do not include the homes where people live, leading to an exposure to environmental tobacco smoke, which is one of the major factors predisposing children to premature death and several hazardous health problems [3].

Focusing the auditory system, smoking and passive smoke exposure are associated with sensorineural hearing loss in children and adults [4]. Experimental studies in mice indicate that passive smoking may potentiate the harmful effects of noise on hearing and disturb the recovery mechanism in the cochlea. In addition, hair cell loss was reported to occur, and outer hair cells seemed to be particularly vulnerable [5,6]. 

Otoacoustic emissions (OAE) allow earlier detection of cochlear auditory dysfunction than the conventional audiogram [7,8]. OAE are sounds vibrations generated by active and non-linear processes of the cochlear outer hair cells as a mechanical feedback. OAE act as an indicator of the cochlear physiological mechanism. This new approach to study the cochlea improves the ways to investigate and interpret hearing loss. Recent findings suggest that the auditory functions in infants of smoking mothers were significantly reduced as measured by OAE [9,10] and auditory brainstem response [11,12,13]. 

The magnitude of each of the adverse effects of smoking and their consequences for medium- and long-term child development have yet to be clearly defined. The objective of the present research was to analyze the effect of tobacco smoke exposure during childhood on cochlear physiology by measuring the transient evoked otoacoustic emissions (TEOAE) response levels.

## 2. Experimental Section

### 2.1. Subjects

We obtained data related to 145 children, 8–10 years old, from a public school in the central region of the city of Sao Paulo, Brazil. All of the children who were invited to participate in the present study had normal hearing test results (including pure tone audiometry, speech audiometry, and tympanometry), no neurological or psychiatric syndromes or disorders (exclusion criteria), and met the following inclusion criteria:

- control group (*n =* 85) having no history of hearing impairment; having no learning disabilities; having no language disorders; not exposed to passive smoking during childhood (urinary cotinine < 5.0 ng/mL);
- tobacco smoke exposure (TSE) group (*n =* 60): exposed to passive smoking during childhood (urinary cotinine ≥ 5.0 ng/mL).

Approval for this study was granted by the local Ethics Committee (Protocol No. CEP FCMSCSP 272/11). Written informed consent was obtained from the parent or guardian of each children tested in this research and the children agreed to participate. 

### 2.2. Procedures

#### 2.2.1. Indirect Cochlear Physiology Evaluation

In the present study, otoacoustic emission levels were used to assess the effects of tobacco exposure on children cochlear physiology. The tests were performed in a sound-treated booth, the TEOAEs being recorded by an Echoport Plus ILO292 otoacoustic emission analyzer (Otodynamics Ltd. Hatfield, UK) coupled to a laptop running the program Echoport Plus ILO292, version 5.61 (Otodynamics Ltd.). A UGS TEOAE probe (Otodynamics Ltd.) and a response time window of 4–20 ms were used. In all children, the TEOAEs were recorded in non-linear mode. For all tests, the clicks were delivered at a peak equivalent sound pressure level (SPL) of 78–83 dB, and a total of 260 sweeps were recorded for both ears.

#### 2.2.2. Nicotine Metabolite Measurements—Cotinine

The main nicotine metabolite excreted in urine is cotinine. Cotinine is used in research as a reliable marker for smoking status and smoking cessation studies. We used an Abnova ELISA kit (Abnova, Taipei, Taiwan) to determine the cotinine concentrations in urine of students. Briefly, the first daily urine sample was collected from the child in his/her house in an appropriate container. In a time no longer than 2 h it was delivery to us and the sample was immediately stored in a −20 °C freezer. The samples were kept frozen till the ELISA assay procedure (1–6 months). From student’s urine samples, levels of cotinine, the main metabolite of nicotine, were measured. It was considered that children were exposed to tobacco if cotinine urine levels were equal or greater than 5.0 ng/mL [14]. 

### 2.3. Data Analysis

TEOAE levels was measured in dB SPL and registered in the right and left ears, respectively, by frequency bands and by overall response, for both groups (TSE and control). 

#### Measurements and Statistical Analysis

The statistical analyses were carried out at the FCMSCSP Center for Applied Statistics. Differences between TSE and control children were analyzed using the Student’s *t*-test by SPSS version 13.0. The level of significance was set at *p =* 0.05. 

## 3. Results and Discussion

A total of 165 children were invited to participate in this research, however only 145 met the inclusion criteria. Twenty were excluded for presenting abnormal results—two with hearing loss and 18 with otitis media. Of them, 16 were in the group of children exposed to tobacco. Tobacco exposure is a serious and increasing global paediatric issue [15]. There is evidence that children whose parents smoke are two to three times greater risk of having recurrent episodes of otitis media [16], which could explain this high difference on the frequency of otitis media comparing students exposed to tobacco or not in our sample.

**Figure 1:**
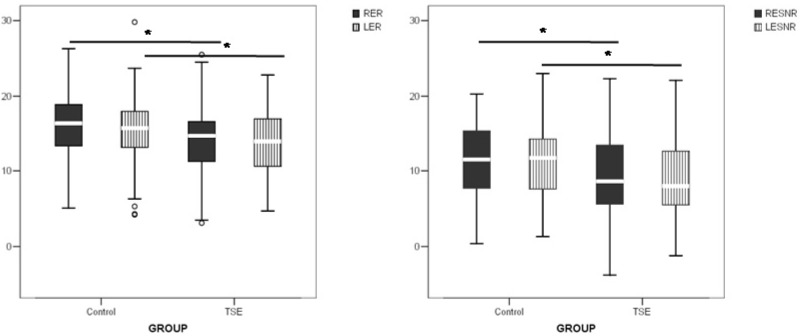
TEOAE response level and signal/noise response level of right (RE) and left (LE) ears of tobacco smoke exposure (TSE) students and their controls. Upper bars indicate comparison between groups per ear. * *p* < 0.05.

Regarding tobacco exposure, on the 145 students included, it was found that 60 were TSE and 85 were not. The ones that showed cotinine levels 0 to 4.9 ng/mL were included in control group. The evaluation of TEOAE on right and left ears of TSE students showed lower response levels and signal noise response levels when compared to controls that were not exposed to tobacco (Figure 1). 

Studies involving smoking and nonsmoking adults have demonstrated that the levels of transient evoked otoacoustic emissions (TEOAEs) were significantly lower in the smokers [17,18,19,20,21,22]. Given the emerging evidence of a relationship between primary tobacco smoking and hearing loss it is reasonable to postulate that a similar association exists between passive smoking and hearing loss. The first study to directly assess the relationship between hearing loss and passive smoking using national data and biomarkers for secondhand exposure in a nationally representative sample of adults was conducted in 2011 [23]. Data collected from non-smoking participants aged 20–69 years were included in the analysis if they had completed audiometric testing, had a valid serum cotinine value, and provided complete smoking, medical co-morbidity and noise exposure histories (*n* = 3,307). It was found that passive smoking is associated with hearing loss in non-smoking adults. Herein we observed a much younger population TSE. As the decrease in TEOAE preceded the hearing loss, and it was found that the children TSE showed a decrease for signal noise response levels of 2.1 dB SPL (95% confidence interval: 1.6–2.5), we hypothesized that the continued exposure to tobacco smoke could lead to a hearing loss.

**Figure 2:**
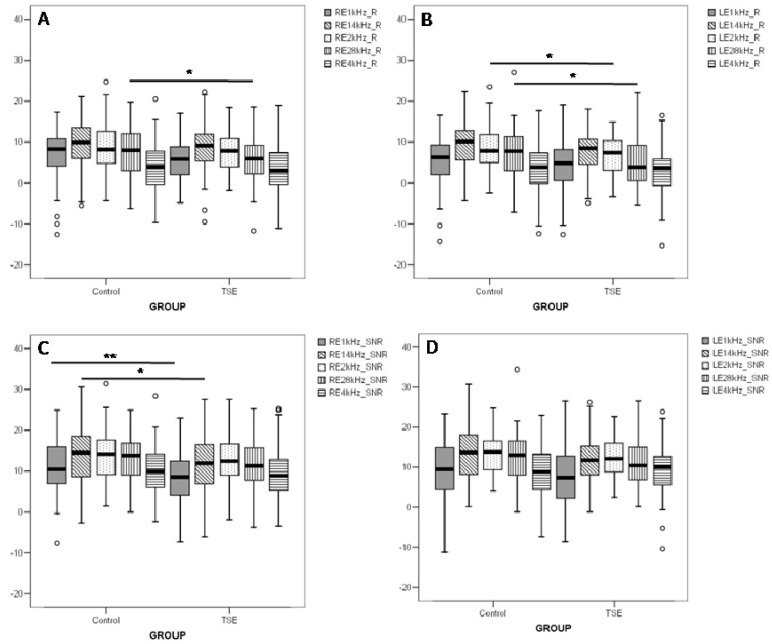
Frequency band TEOAE response level and signal/noise response level of right (RE) and left (LE) ears of tobacco smoke exposure (TSE) students and their controls. Upper bars indicate comparison between groups per ear. * *p* ≤ 0.05 and ** *p* ≤ 0.01.

After a general analysis of TEOAE responses, different frequency bands were decomposed by fast Fourier transform and are shown in Figure 2. Statistical differences were found in the reduced response levels of TSE group compared to controls in: (a) the 2.8 kHz frequency band for both ears and (b) the 2.0 kHz frequency band for the left ear (Figure 2(A,B)). In the signal/noise response levels (Figure 2(C,D)) a statistical difference was found for the right ear in the frequency bands of 1.0 kHz and 1.4 kHz (Figure 2(C)). These statistical differences obtained when decomposing the frequencies could partially explain the differences seen in the general responses. The whole difference could be to the fact that each frequency band mean in response levels and signal/noise response levels was lower on the TSE group when comparing to control, but 4 kHz on signal/noise response levels for the left ear.

Cigarette smoking has been shown to decrease hearing levels for frequencies in the 0.25–8 kHz range [24]. Regarding the otoacoustic emissions, the smokers group showed a lower response level in the frequencies of 1 kHz in both ears and 4 kHz in the left ear [19]. Comparisons between exposed newborns to control group revealed statistically significant decreases of TEOAEs amplitudes at 4 kHz [10].

A markedly reduced response level observed in the TSE children is consistent with previous reports in neonates. Prenatal exposure to nicotine seems to have adverse effects on hearing, which are detectable as early as in the neonatal phase as determined by otoacoustic emissions testing [9,10,11]. TSE is a chronic exposure, probably during a whole child’s life. The TEOAE is an indirect measure of cochlear physiology which appears reduced in the TSE children. A reduced cochlear physiology during all a child’s lifetime, comprising the critical period of hearing abilities development, could lead to a progressive auditory disorder. 

Maternal smoking has also been shown to affect child development. Some studies have suggested that maternal smoking during pregnancy can lead to intellectual delays, most likely caused by central nervous system impairment [25], or can negatively affect language ability through underlying physiologic mechanisms (e.g., the outer hair cells in the ear), thus leading to poorer performance on auditory processing tasks [26], temporal auditory processing [27], auditory brainstem responses [11,12,13] and attention deficit hyperactivity disorder [28]. The effects of maternal smoking during pregnancy on infants’ speech processing ability were previously investigated by event-related potentials (ERPs). Infants of smoking mothers initially demonstrated no hemisphere differences, and after a delayed time period their ERPs indicated an inconsistent pattern of hemisphere differences, with left hemisphere amplitudes larger for some speech sounds and smaller for others. There were notable group differences in the speed of the brain responses and the number of discriminations between speech sounds. These findings indicate that prenatal exposure to tobacco smoke in otherwise healthy babies is linked with significant changes in brain physiology associated with basic perceptual skills that could place the infant at risk for later developmental problems. Infants born to smoking mothers may also be more likely to be exposed to environmental tobacco smoke, thus further increasing their risk of adverse developmental outcomes [29]. 

The loss in cochlear physiology here detected in 8–10 year old students is one of impairment of the sensorial system. Other indirect effects of nicotine exposure could assume synergistic roles in affecting the neural systems. The anorexigenic effects of nicotine are associated with poor nutritional status of mothers and fetuses. Low birth weight (due to prenatal exposure to nicotine or otherwise) seems to be directly related to neurological disorders such as attention deficit hyperactivity disorder and hyperactivity [28,30,31,32].

Questions still remain about the relative roles of prenatal *vs*. postnatal nicotine exposure and the potential of genetic and social factor confounders. The consistency of findings across studies is, however, highly suggestive of a causal relationship between environmental tobacco exposure and adverse behavioral and cognitive outcomes in children [33]. An important datapoint comes from the Global Youth Tobacco Survey that indicates that almost half of the children who had never smoked were exposed to second-hand smoke at home (46.8%) or outside the home (47.8%) [34]. Efforts should be intensified to increase the knowledge, promotion, and referral to effective interventions to help parents and pregnant smokers to quit smoking.

## 4. Conclusions

Tobacco smoke exposure had a negative effect on cochlear function of 8–10 year old students, as assessed by otoacoustic emissions testing. The mean loss on tobacco smoke exposure children was 2.1 dB SPL. These results have important implications for the damage to the cochlear structures and indicate a possible loss in hearing and hearing ability development. 
